# Ultralow Interfacial Thermal Resistance of Graphene Thermal Interface Materials with Surface Metal Liquefaction

**DOI:** 10.1007/s40820-022-00979-2

**Published:** 2022-12-09

**Authors:** Wen Dai, Xing-Jie Ren, Qingwei Yan, Shengding Wang, Mingyang Yang, Le Lv, Junfeng Ying, Lu Chen, Peidi Tao, Liwen Sun, Chen Xue, Jinhong Yu, Chengyi Song, Kazuhito Nishimura, Nan Jiang, Cheng-Te Lin

**Affiliations:** 1 Key Laboratory of Marine Materials and Related Technologies, Zhejiang Key Laboratory of Marine Materials and Protective Technologies, Ningbo Institute of Materials Technology and Engineering (NIMTE), Chinese Academy of Sciences, Ningbo, 315201 People’s Republic of China; 2 Center of Materials Science and Optoelectronics Engineering, University of Chinese Academy of Sciences, Beijing, 100049 People’s Republic of China; 3 Institute of Advanced Technology, Shandong University, Jinan, 250100 People’s Republic of China; 4 Ningbo Institute of Materials Technology and Engineering (NIMTE), Chinese Academy of Sciences, Ningbo, 315201 People’s Republic of China; 5 The State Key Laboratory of Metal Matrix Composites, School of Materials Science and Engineering, Shanghai Jiao Tong University, 800 Dong Chuan Road, Shanghai, 200240 People’s Republic of China; 6 Advanced Nano-Processing Engineering Lab, Mechanical Systems Engineering, Kogakuin University, Tokyo, 192-0015 Japan

**Keywords:** Vertically aligned graphene, Liquid metal, Surface modification, Thermal interface materials

## Abstract

Developing advanced thermal interface materials (TIMs) to bridge heat-generating chip and heat sink for constructing an efficient heat transfer interface is the key technology to solve the thermal management issue of high-power semiconductor devices. Based on the ultra-high basal-plane thermal conductivity, graphene is an ideal candidate for preparing high-performance TIMs, preferably to form a vertically aligned structure so that the basal-plane of graphene is consistent with the heat transfer direction of TIM. However, the actual interfacial heat transfer efficiency of currently reported vertically aligned graphene TIMs is far from satisfactory. In addition to the fact that the thermal conductivity of the vertically aligned TIMs can be further improved, another critical factor is the limited actual contact area leading to relatively high contact thermal resistance (20–30 K mm^2^ W^−1^) of the “solid–solid” mating interface formed by the vertical graphene and the rough chip/heat sink. To solve this common problem faced by vertically aligned graphene, in this work, we combined mechanical orientation and surface modification strategy to construct a three-tiered TIM composed of mainly vertically aligned graphene in the middle and micrometer-thick liquid metal as a cap layer on upper and lower surfaces. Based on rational graphene orientation regulation in the middle tier, the resultant graphene-based TIM exhibited an ultra-high thermal conductivity of 176 W m^−1^ K^−1^. Additionally, we demonstrated that the liquid metal cap layer in contact with the chip/heat sink forms a “liquid–solid” mating interface, significantly increasing the effective heat transfer area and giving a low contact thermal conductivity of 4–6 K mm^2^ W^−1^ under packaging conditions. This finding provides valuable guidance for the design of high-performance TIMs based on two-dimensional materials and improves the possibility of their practical application in electronic thermal management.

**Supplementary Information:**

The online version contains supplementary material available at 10.1007/s40820-022-00979-2.

## Highlights

- A three-tiered thermal interface materials was proposed with the through-plane thermal conductivity up to176 W m^−1^ K^−1^ and contact thermal resistance as low as 4–6 K mm^2^ W^−1^ (double sides).
- The liquid metal acts as a buffer layer to connect vertically aligned graphene with the rough heater/heat sink, improving effective contact thermal conductance by more than an order of magnitude.

**Supplementary Information:**

The online version contains supplementary material available at 10.1007/s40820-022-00979-2.

## Graphical abstract

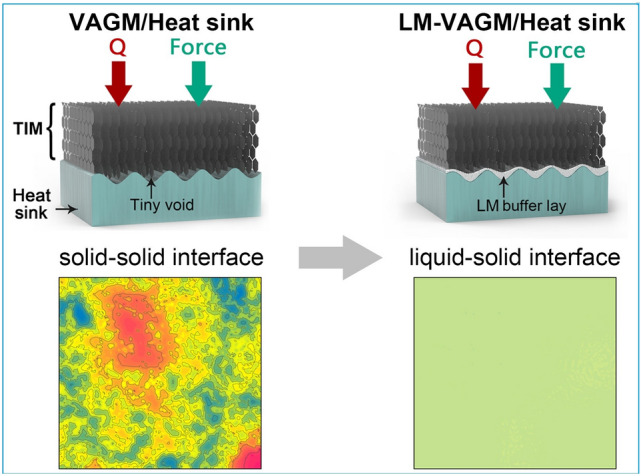

## Introduction

The past half-century has witnessed the rapid development of semiconductor chips, mainly including the continuous reduction in feature size of silicon-based chips and the widespread use of high frequency/power wide-bandgap semiconductor chips such as gallium nitride (GaN) and silicon carbide (SiC) [1–3]. The progress of semiconductor technology, while giving electronic devices higher efficiency and more functionality, has also resulted in a dramatic increase in the power density of the chips, giving rise to serious heat dissipation issues [4–6]. Accordingly, researchers continue to propose various advanced thermal design solutions to meet the challenges. At present, it is generally believed that effective thermal design depends not only on the reasonable system architecture but also on the thermal conductivity of the applied packaging components, including chips (heater), circuit board, substrate, heat sink, and thermal interface materials (TIMs) [7–9]. Among them, the role of soft TIMs is to connect other rigid components to enhance the interfacial thermal conductance between them by excluding non-flowing air (thermal conductivity: ≈ 0.026 W m^−1^ K^−1^) in the microgaps formed by their mating interface [10–12]. In practical application, to maximize the heat transfer efficiency, TIMs not only need to have high through-plane thermal conductivity (κ_⊥_), but also need to form a low thermal contact resistance (*R*_contact_) with the paired rigid heater/heat sink [13, 14]. In general, one of the essential routes for achieving low *R*_contact_ is to ensure good compressibility and easy deformation of the TIMs to adequately fill the interfacial microgaps between the heater and heat sink under normal packaging conditions [15]. Currently, the most common TIMs are basically elastic polymer composites consisting of thermally conductive fillers (such as Al_2_O_3_, AlN, and Cu/Ag particles) and silicone matrix (generally, polydimethylsiloxane, PDMS) [16–18]. Due to the trade-off between thermal conductivity and compressibility in this filler-matrix system, such conventional TIMs usually have a limited κ_⊥_ of 1–5 W m^−1^ K^−1^ and a high *R*_contact_ of 30–60 Kmm^2 ^W^−1^ [10–20]. On this basis, conventional TIMs have become increasingly challenging to handle the thermal management issues of today’s high frequency/power chips, leading to an urgent need to develop high-performance TIMs for advanced thermal designs.

Since graphene was reported in 2004 and exhibited ultrahigh thermal conductivity and easy implementation of structure modulation from microscopic to macroscopic, numerous studies have focused on developing graphene-based TIMs to solve the interfacial heat transfer issue [21, 22]. Based on the fact that the thermal conductivity of graphene is highly anisotropic with excellent heat transfer performance only along the basal-plane direction (basal-plane: 3500–5300 W m^−1^ K^−1^; out-of-plane: 2 W m^−1^ K^−1^), the most effective structure design for graphene-based TIMs is to modulate graphene to form vertical alignment [23–25]. In this case, the basal-plane of graphene is consistent with the heat transfer direction of the TIMs, which can maximize the utilization of its ultrahigh basal-plane thermal conductivity. Currently, a widely reported method for preparing vertically aligned graphene-based TIMs is the directional assembly of graphene nanosheets to form a vertically aligned and porous structure of graphene, followed by refilling the polymer matrix [26–29]. Based on this primary route, Yu et al. utilized the liquid crystal orientation properties of graphene oxide (GO) and combined the graphitization post-treatment to obtain a graphene/polymer composite (19 vol%) with a remarkably high κ_⊥_ of 35.5 W m^−1^ K^−1^ [30]. Besides, the vertically aligned graphene-based TIMs can also be readily prepared by the secondary assembly of graphene paper [24, 25, 31]. For example, Zeng et al. stacked the polybutadiene-modified graphene paper (40 wt%) layer-by-layer, then sliced it along the thickness direction to obtain a TIM with the κ_⊥_ of 83.7 W m^−1^ K^−1^ [32]. Finally, the recently emerging direct growth of vertical graphene arrays by plasma-enhanced chemical vapor deposition (PECVD) is also an effective method for preparing vertically aligned graphene-based TIMs [33–35]. Xu et al. demonstrated that the resultant TIM obtained by adding 8.6 wt% vertical graphene arrays to PDMS has a κ_⊥_ of 34.3 W m^−1^ K^−1^ with good compressibility [36]. Based on the above progress, various vertically aligned graphene-based TIMs reported so far have made great breakthroughs in κ_⊥_ compared with conventional TIMs, but their actual heat dissipation efficiency cannot still meet expectations in the packaging conditions. In addition to the fact that the κ_⊥_ of currently vertically aligned graphene TIMs can still be further improved, another critical factor is the relatively high *R*_contact_ (20–30 K mm^2^ W^−1^) of the mating interface formed by the vertical graphene structure in contact with the heater/heat sink [27, 31, 37]. This is mainly because the graphene edges constituting the surface of the vertically aligned graphene structure are discretely distributed and have a height difference. It is difficult for graphene edges to fully deform to fit a rough surface under the normal packaging pressure, resulting in a limited actual contact area between the TIM and heater/heat sink. Such an insufficient contact state severely suppresses the efficient heat transfer across the mating interface, which is a common problem faced by almost all vertically aligned graphene-TIMs. Therefore, toward the practical application, in addition to rational structural modulation for continuing to improve the κ_⊥_ of vertically aligned graphene TIMs, further efforts to optimize the contact state between the TIM and the heater/heat sink for reducing the *R*_contact_ must be addressed.

Herein, focusing on this issue, a three-tiered TIM design concept was proposed and implemented in this work. We first adopted a mechanical orientation strategy to prepare vertically aligned graphene monoliths (VAGM) and then carried out thermal evaporation of micrometer-thick liquid metal onto its upper and lower surfaces to obtain a sandwich-structured TIM. Based on rational structural design, the resultant liquid metal-modified VAGM (LM-VAGM) exhibited an ultra-high κ_⊥_ of 176 W m^−1^ K^−1^ while having good compressibility. Besides, the surface modification of liquid metal with excellent deformability greatly improves the gap-filling performance of LM-VAGM in contact with a rough heater/heat sink, exhibiting a much low *R*_contact_ of 4–6 K mm^2^ W^−1^ under actual packaging conditions. The comparative thermal management performance verification between the LM-VAGM and state-of-the-art commercial TIMs demonstrated the superior interface heat transfer efficiency of our proposed TIM for cooling electronic devices.

## Experimental Section

### Materials

Graphene sheets were purchased from Ningbo Morsh Technology Co., Ltd. (China) with an average thickness of 10.6 nm and a lateral size of 5.4 nm. The corresponding characterizations of graphene sheets can be found in our previous works [38]. Anhydrous ethanol was obtained from Sinopharm Chemical Reagent Co., Ltd. (China) and used without further purification.

### Preparation of VAGM

The preparation process of VAGM is mainly divided into three steps. Firstly, a conventional vacuum filtration of graphene/ethanol dispersion (2 mg mL^−1^) was performed to prepare a graphene paper. In order to obtain a graphene paper with a diameter of 28 cm, the filtration equipment was specially processed and customized with a porous polytetrafluoroethylene (PTFE) membrane as filter medium (porous size: 0.22 μm). We then employed a cold pressing followed by thermal treatment (800 °C/2 h and 2800 °C/2 h) to adjust the density (1.52 g cm^−3^) and in-plane thermal conductivity (553 W m^−1^ K^−1^) of the graphene paper for suiting subsequent processing requirements. Subsequently, the as-prepared graphene paper was pasted onto a pre-stretched elastomer (VHB 4910, 3 M, USA) using a self-developed eight-axis stretching device to control an extension ratio of 250%. Then, we controlled the tape to shrink slowly, by which part of the graphene paper would be detached from the elastomer to form a crumpled graphene paper. After removing the elastomer by ethanol soaking for several hours, a biaxial compression operation (compression ratio of 40%) was carried out to deform the crumpled graphene paper into a hierarchically structured graphene monolith (HSGM) with the size of 2.5 cm × 2.5 cm × 2 mm and a density of ≈ 0.63 g cm^−3^. Finally, we used precision mechanical polishing to remove the upper and lower surface layers of HSGM. And the VAGM was obtained by using the high-temperature hydrogen plasma (850 °C for 30 min) for etching away the polishing residue. To generate the stable hydrogen plasma, the output power of microwave plasma CVD (MPCVD) is 3.6 kW, with the corresponding hydrogen flow and a chamber pressure of 400 sccm and 10.8 kPa, respectively.

### Preparation of LM-VAGM

The preparation process of LM-VAGM is mainly divided into two steps. Firstly, the electron beam evaporation of the titanium/gold (Ti/Au) on the upper and lower surfaces of VAGM was performed. The Ti layer with a thickness of 20 nm was deposited using the DZS 500 E-beam system at a rate of 0.35–0.4 Å s^−1^ with the ultimate pressure lower than 1E-3Pa. After depositing Ti as a buffer layer, we proceeded to deposit a 50 nm Au layer, with the rate of 0.35–0.4 Å s^−1^ at room temperature. Then, the vacuum thermal evaporation was carried out to deposit the liquid metal gallium on the upper and lower surfaces of Ti/Au-coated VAGM. The heating current applied to the tungsten evaporation boat at the start of deposition was 180 A and the pressure in the chamber was 3E-3Pa. In order to form a gallium layer with a thickness of 4–5 μm for obtaining the LM-VAGM, the deposition time is about 4 min.

### Characterization

The microstructure of all samples was observed using field emission scanning electron microscopy (SEM, Quanta FEG250, FEI, USA). The stress–strain curves of VAGM and LM-VAGM were obtained based on a universal testing machine (UTM, model 5567A, Instron, USA), in which the sample size is 1 cm × 1 cm × 0.8 mm, and the loading rate is 0.1 mm min^−1^. The compressive modulus of VAGM and LM-VAGM can be obtained by calculating the average value of the tangent modulus (*E* = d*σ*/d*ε*) in the range of 0–10% strain, where σ is the compressive stress, and ε is the corresponding strain. The thermal conductivity of the samples was measured with an LFA 467 NanoFlash apparatus (Netzsch, Germany). The thermal resistance was measured using a Longwin 9389 thermal conductivity tester. The IR photos were captured by using an infrared camera (Fluke, Ti400, USA). To ensure that the contact states of LM-VAGM and tungsten with the heating plate and the IR emissivity of the two samples were consistent, we sprayed a dense graphite layer (5 μm) on the upper and lower surfaces of LM-VAGM and tungsten. The thermal cycling test LM-VAGM was performed by alternately altering the heater temperature between 30 °C (40 W heating for 10 s) and 133 °C (400 W heating for 10 s), with the cycling frequency of 0.05 Hz.

## Results and Discussion

### Preparation and Structural Characterization of LM-VAGM

**Fig. 1:**
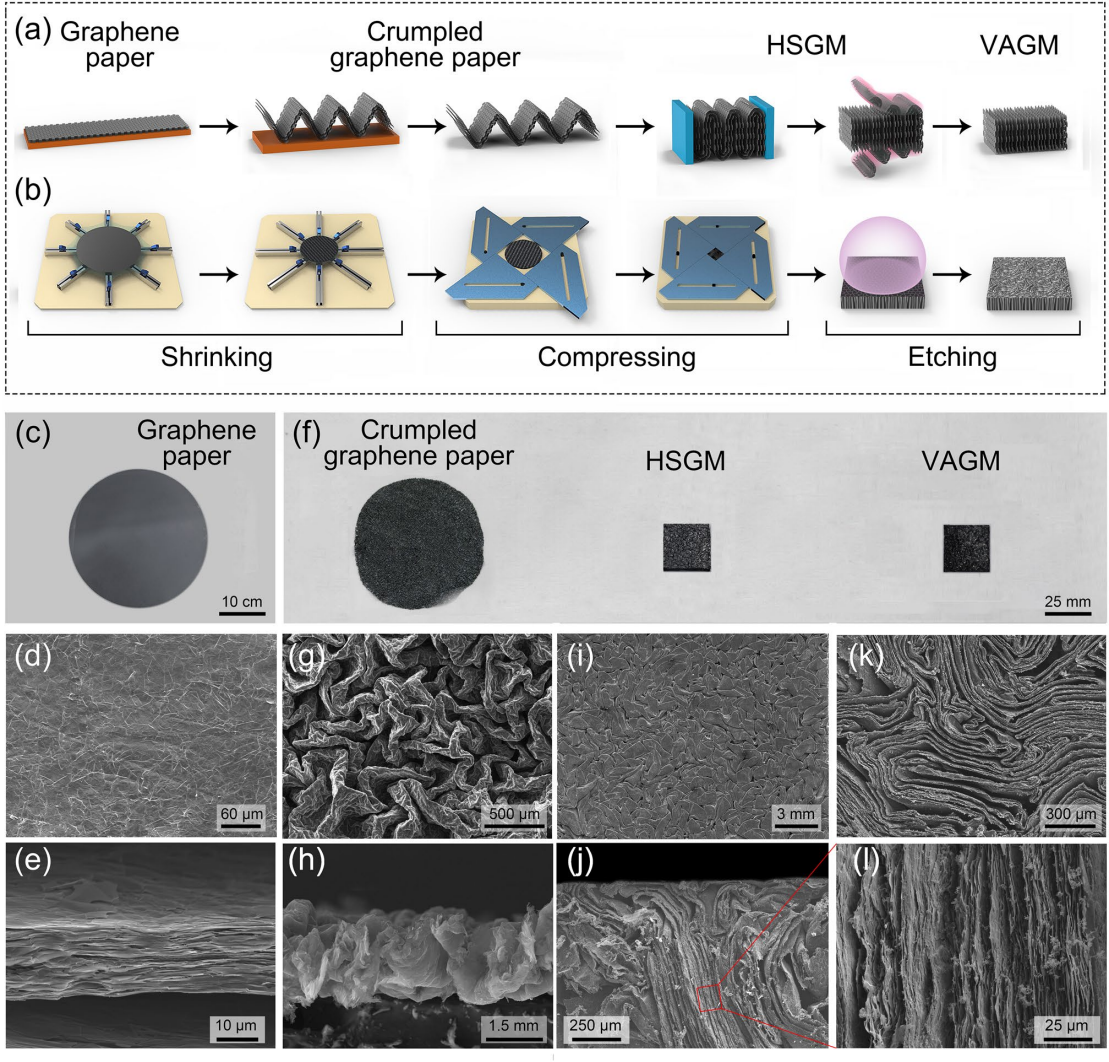
Schematic illustrating **a** structure modulation concept and **b** preparation process of VAGM. **c** Photograph, **d** cross-sectional and **e** top-view SEM images of graphene paper. **f** Photograph of crumpled graphene paper, HSGM and VAGM, with the corresponding cross-sectional and top-view SEM images showing in (**g**–**l**)

The preparation process of the sandwich-structured LM-VAGM was divided into two steps: the first fabrication of VAGM as the middle layer and the subsequent surface modification to form a cap layer using liquid metal (LM). The design concept of VAGM is to use graphene paper as the raw material, followed by a mechanical orientation process to convert the horizontally arranged graphene inside the graphene paper into a vertical alignment. The structural transition and preparation process from graphene paper to VAGM is illustrated in Fig. 1a, b, respectively, and a detailed description of the whole procedure can be found in the Experimental Section. The graphene paper with a diameter of ≈ 28 mm and a thickness of ≈ 15 μm was obtained via the filtration of graphene nanosheets, combined with hot-pressing and subsequent graphitization treatment (Fig. 1c). In Fig. 1d, e, the graphene sheets inside the graphene paper are stacked layer-by-layer along the horizontal direction. To modulate the orientation of the graphene sheets, we first pasted the as-prepared graphene paper on the pre-stretched elastomer, then controlled the elastomer shrinkage slowly through a self-developed eight-axis stretching device (Fig. 1b). Since graphene paper has almost no horizontal compressibility, during the shrinkage of the elastomer, part of the graphene paper would be detached from the elastomer, forming a crumpled texture, as shown in Fig. 1f–h. After removing the elastomer by ethanol soaking, a biaxial compression operation was carried out to deform the obtained crumpled graphene paper into a hierarchically structured graphene monolith (HSGM). In Fig. 1i, j, the middle layer of HSGM is composed of vertically aligned graphene, and its upper and lower surfaces are covered with a layer of horizontally aligned graphene, which is formed by flattening the peaks and valleys of crumpled graphene paper. A more detailed characteristic structure analysis of the HSGM can be found in Fig. S1. Finally, a precise mechanical polishing followed by high-temperature hydrogen plasma etching (850 °C for 30 min) was performed to remove the horizontal graphene layers on the upper and lower surfaces of the HSGM to obtain the VAGM. In contrast to the mosaic-like surface morphology of HSGM (Fig. 1i), the VAGM surface consists entirely of vertically aligned and closely packed graphene edges, as shown in Fig. 1k. As a result, by combining mechanical orientation and subsequent accessory processing, we obtained a VAGM composed entirely of vertically aligned graphene (Fig. 1l) as the middle layer of the proposed LM-VAGM.

**Fig. 2:**
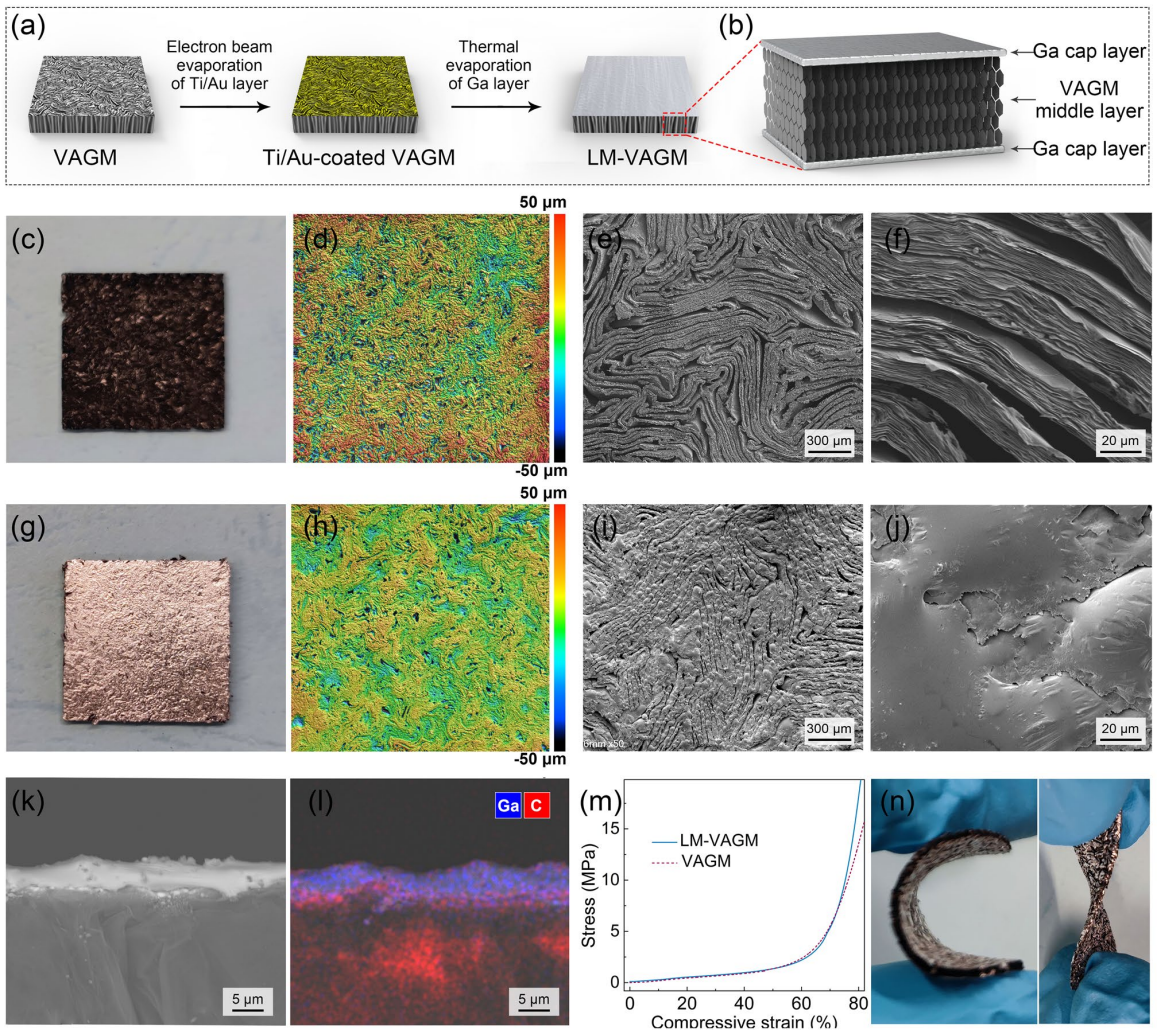
Schematic illustrating **a** preparation process from VAGM to LM-VAGM and **b** structural feature of LM-VAGM. **c** Photograph, **d** surface topographic and **e**, **f** top view SEM images of VAGM. **g** Photograph, **h** surface topographic and **i**, **j** top-view SEM images of LM-VAGM. **k** Typical cross-sectional SEM image of LM-VAGM with the corresponding element mapping showing in **l**. **m** Compressive stress–strain curves of VAGM and LM-VAGM. **n** The obtained LM-VAGM showing good bendability

When the VAGM was ready, a post-surface modification based on liquid metal gallium was carried out to prepare LM-VAGM (Fig. 2a). Gallium was chosen because of its suitable melting point of 29.8 °C, by which gallium can generally remain solid at room temperature, or it is easy to construct an environment that keeps gallium solid. Besides, the operating temperature of consumer electronic chips is usually over 40 °C. At this temperature, the gallium at the LM-VAGM surface can become liquid to build an efficient heat transfer interface between the TIM and the substrate (heater/heat sink). In order to deal with the poor wettability between gallium and graphene, the upper and lower surfaces of VAGM were pretreated by an electron beam evaporation of the titanium/gold (Ti/Au) layer (Ti: 20 nm, Au: 50 nm). Subsequently, the resultant LM-VAGM can be obtained by decorating micrometer-thick liquid gallium on the surface of Ti/Au-coated VAGM based on a vacuum thermal evaporation process, as the corresponding structure schematically illustrated in Fig. 2b. Figure 2c–j expatiated the surface morphology transition from pristine VAGM to LM-VAGM, and on a macroscopic scale, the surface color of the sample changes from black (Fig. 2c) to silvery white (Fig. 2g) after the modification of the gallium. The comparison between Fig. 2d–f and Fig. 2h–j indicates that gallium completely covers the exposed vertical graphene edges, and the surface roughness is also reduced from 17.2 μm for VAGM to 6.7 μm for LM-VAGM. In Fig. 2k, l, the cross-sectional SEM image and the corresponding EDS mapping of LM-VAGM indicate that the gallium is only distributed on the surface of the sample to form a cap layer with a thickness of 4–5 μm. Accordingly, the interior of LM-VAGM still remained a porous and vertically aligned graphene structure, which endows the sample with comparable compressibility to VAGM (Fig. 2m). As a result, the LM-VAGM with a low compression modulus of 2.25 MPa exhibits excellent flexibility and bendability, as shown in Fig. 2n.

### Thermally Conductive Performance of LM-VAGM

**Fig. 3:**
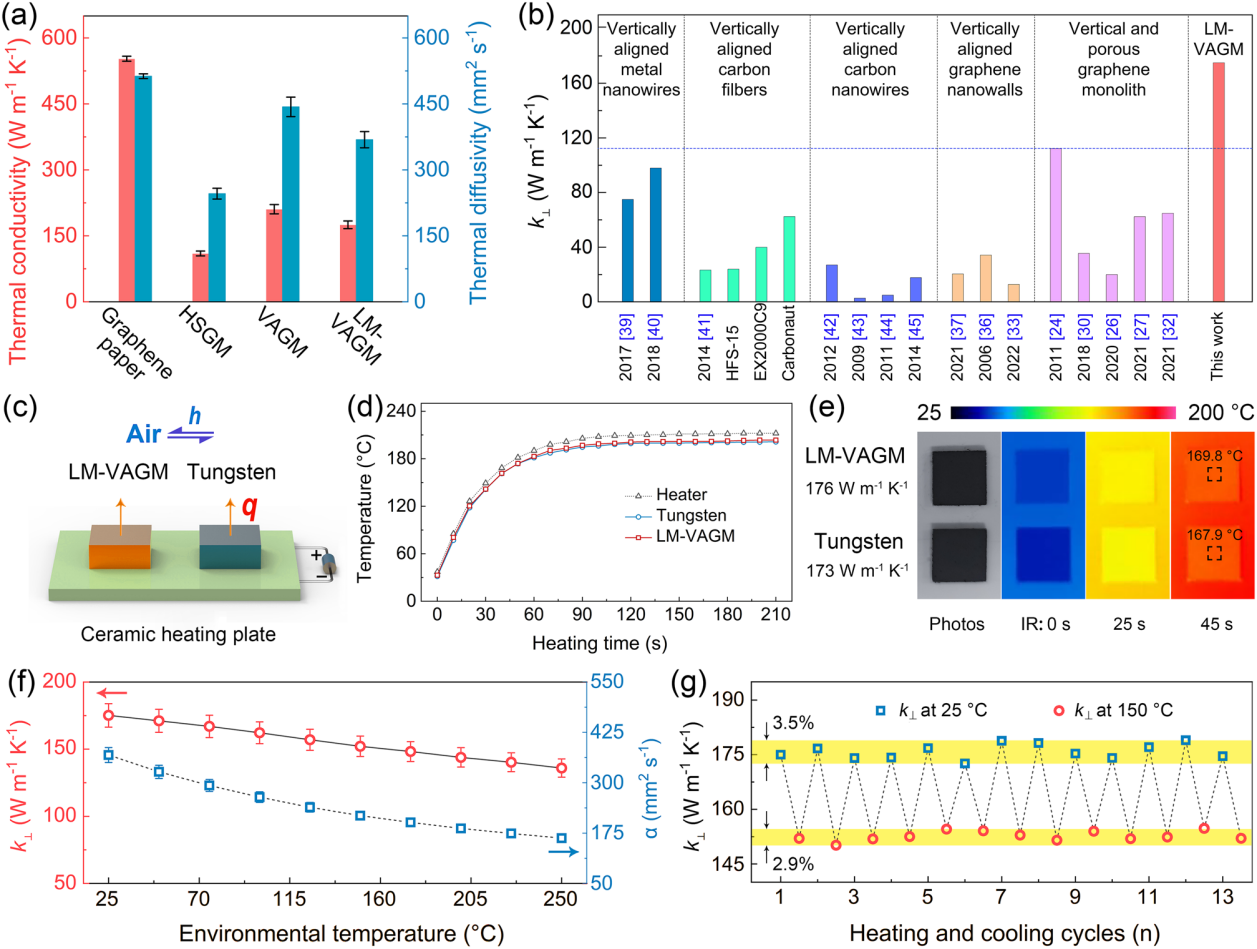
**a** In-plane thermal conductivity of graphene paper and through-plane thermal conductivity (κ_⊥_) of HSGM, VAGM and LM-VAGM. **b** Comparison of the κ_⊥_ of LM-VAGM with the reported thermally conductive materials based on vertically aligned structure. **c** The test platform for demonstrating the heat transfer capacity along the through-plane direction, with the resultant surface temperature evolution and the corresponding IR images shown in **d** and **e**, respectively. The κ_⊥_ of LM-VAGM as a function of **f** environmental temperature and **g** high/low-temperature cycle

Based on the characteristic sandwich structure with vertically aligned graphene as the middle layer, we proposed that the LM-VAGM can have excellent heat transfer performance along the vertical direction. Accordingly, the intrinsic κ_⊥_ of the LM-VAGM was measured using the laser flash technique (ASTM E1461), with the detailed measurement process shown in Section S1. And to in-depth understand the structure–function relationship between graphene arrangement and heat transfer efficiency, the heat conduction properties of the starting graphene paper, HSGP and VAGM were also investigated. In Fig. 3a, based on the horizontal and layer-by-layer stacked graphene structure, the thermal conductivity of the graphene paper exhibits obvious anisotropy, with the value of 553 and 2.4 W m^−1^ K^−1^ along the vertical and horizontal directions, respectively. When the mechanical orientation process was performed, the horizontal heat transfer property of graphene paper was shifted to the vertical direction, endowing the obtained HSGP and VAGM with preferred κ_⊥_ of 109 and 211 W m^−1^ K^−1^, respectively. The superior κ_⊥_ of VAGM compared to that of HSGM can be attributed to the removal of the horizontal graphene layers with low κ_⊥_ on its upper and lower surfaces. After the liquid metal gallium was further modified on the VAGM surface as the cap layer, the resultant LM-VAGM exhibits a slightly lower κ_⊥_ of 176 W m^−1^ K^−1^ compared to that of VAGM. Nevertheless, this thermal conductivity achieved is still higher than that of most thermally conductive materials based on various vertical structures reported so far, including vertically aligned metal nanowires [39, 40], carbon fibers [41], carbon nanowires [42–45], graphene nanowalls [33, 36, 37], and graphene monolith [24, 26, 27, 30, 32], as shown in Fig. 3b and Table S1.

In order to visually demonstrate the high κ_⊥_ of LM-VAGM, a comparative test on heat transfer capacity between LM-VAGM and pure tungsten (*W*, ≈ 173 W m^−1^ K^−1^) was performed. As the testing platform shown in Fig. 3c, we first cut LM-VAGM and tungsten into the same size (10 × 10 × 0.8 mm^3^), and sprayed a dense graphite layer (5 μm) on both their upper and lower surfaces, then placed them on a ceramic heating plate (55 W). The purpose of spraying the graphite layer is to make the contact state of the two samples and the substrate consistent, and also to ensure that the infrared emissivity of their upper surfaces is the same, so that the surface temperature changes can be monitored simultaneously with a commercial infrared (IR) camera. In Fig. 3d, e, when the two samples are heated simultaneously, the temperature evolution of them is almost the same, and the surface temperature of LM-VAGM can even stay 1–2 °C higher than that of tungsten during the steady state, determining its superior heat transfer performance along the through-plane direction. Besides, toward the actual use as TIM, the environmental temperature-dependent κ_⊥_ of LM-VAGM was investigated. In Fig. 3f, the κ_⊥_ exhibits a linear decrease trend when the temperature increases from 25 to 250 °C, mainly due to the Umklapp scattering mechanism of crystalline materials, consistent with previously reported graphene-based materials [46, 47]. Nevertheless, the κ_⊥_ of LM-VAGM is still higher than 150 W m^−1^ K^−1^ from room temperature to 150 °C, showing excellent heat transfer performance in the normal device operating temperature range. Figure 3g presents the cyclic heating/cooling κ_⊥_ of LM-VAGM, in which the κ_⊥_ deviation is as low as 3.5% and 2.9% at 25 and 150 °C, respectively, demonstrating the excellent thermal cycling reliability of the LM-VAGM during long-range operation.

**Fig. 4:**
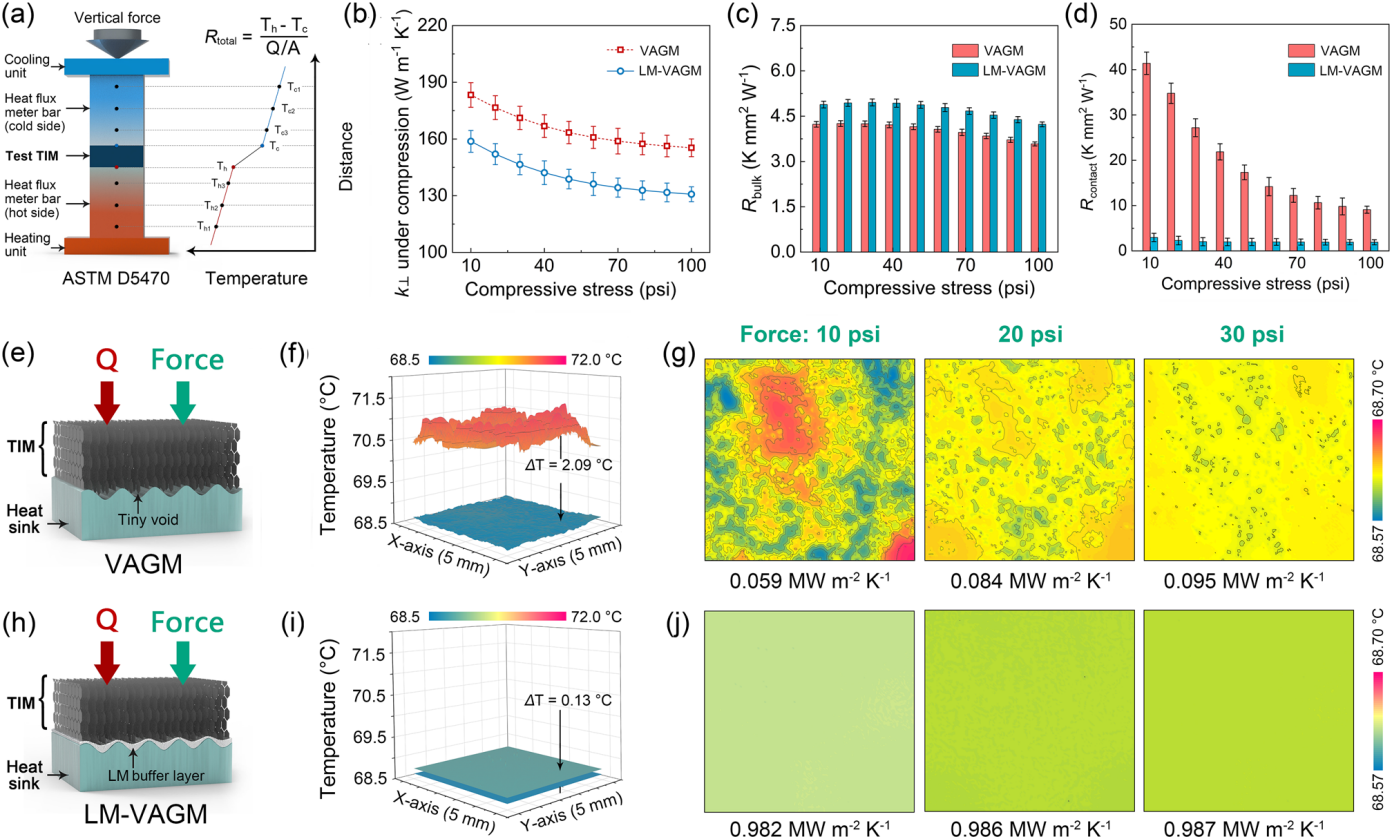
**a** Schematic illustrating the measurement principle of the TIM performance based on a modified ASTM D5470 method, with the obtain κ_⊥_, *R*_bulk_ and *R*_contact_ of the VAGM and LM-VAGM showing in **b**–**d**. The original thickness of the VAGM and LM-VAGM is 800 μm. **e** Schematic illustrating the contact status of VAGM with the rough surface of heat sink. **f** Simulated temperature profiles at the VAGM/heat sink mating interface. **g** Upper surface temperature distribution on the heat sink for the VAGM/heat sink mating interface. **h**–**j** The same cases for the LM-VAGM in contact with heat sink

Generally, for TIMs, the most critical performance is the total thermal resistance exhibited under the actual packaging conditions. When the TIM is connected to the heater and the heat sink under normal packaging pressure, the components of the total thermal resistance (*R*_total_) are shown in Eq. (1):

1 $${R}_{\mathrm{total}}={R}_{\mathrm{bulk}}+{R}_{\mathrm{contact}}=\frac{\text{BLT}}{{\upkappa }_{\perp }}+{(R}_{\mathrm{heater}/\mathrm{TIM}}+{R}_{\mathrm{TIM}/\mathrm{heat sink}})$$

where *R*_bulk_ is the bulk thermal resistance of the TIM, which is the ratio of bond line thickness (BLT) and the intrinsic κ_⊥_; *R*_contact_ is the contact thermal resistance of the TIM, equal to the sum of the thermal boundary resistances generated when the TIM is in contact with the heater (*R*_heater/TIM_) and the heat sink (*R*_TIM/heat sink_), respectively. Based on Eq. (1), high-performance TIMs with low *R*_total_ require not only high κ_⊥_ but also low *R*_contact_ under the actual packaging conditions. Accordingly, we used the steady-state heat-flow method to analyze the κ_⊥_ and *R*_contact_ of LM-VAGM under compression, and compared it with VAGM. This method is based on the ASTM D5470 standard (Fig. 4a), which is a common route for evaluating interfacial heat transfer properties of TIMs. The detailed measurement principles and calculation procedures can be found in Supporting Information (Section S3) with the results shown in Fig. 4b–d. In Fig. 4b, as the applied pressure increases, the κ_⊥_ of LM-VAGM (131–159 W m^−1^ K^−1^) shows a downward trend and is always lower than that of VAGM (155–183 W m^−1^ K^−1^), consistent with the test results shown in Fig. 3a. Since LM-VAGM and VAGM have the same original thickness (800 μm) and similar compressibility, leading to almost the same BLT, the calculated *R*_bulk_ of the LM-VAGM is slightly higher than that of VAGM by 13–15% in the pressure range from 10 to 100 psi (LM-VAGM: 4.2–5.0 K mm^2^ W^−1^, VAGM: 3.6–4.3 K mm^2^ W^−1^, in Fig. 4c). However, in contrast to the approximate κ_⊥_ and *R*_bulk_ of the two samples, Fig. 4d indicates that the *R*_contact_ of LM-VAGM is much lower than that of VAGM under the compression. Especially at a low packaging pressure of 10–30 psi, the LM-VAGM exhibited a *R*_contact_ of 4.1–5.9 K mm^2^ W^−1^, which is about 7% of VAGM (54–82 K mm^2^ W^−1^).

We proposed that the difference in *R*_contact_ of the two TIMs is due to the significantly different contact states of VAGM and LM-VAGM with the heater/heat sink. In order to in-depth elaborate on the interfacial heat transfer mechanism, the numerical simulation based on the multi-block lattice Boltzmann method was conducted to calculate the temperature distribution of the mating surface when the VAGM and LM-VAGM were in contact with the heat sink, respectively. The detailed model implementation can be found in Section S4, with the corresponding results shown in Fig. 4e–j. As schematically illustrated in Fig. 4e, the surface of VAGM was composed of discretely distributed and vertically aligned graphene edges with irregular height. It is difficult for the VAGM surface to deform enough to perfectly match the rough surface of the heat sink under a normal packaging pressure, inevitably creating micro- and nano-scale tiny voids at their mating interface with limited contact area. In Fig. 4f, such a contact state seriously hinders the heat transfer efficiency across the VAGM/heat sink interface, resulting in a large temperature difference (2.09 °C) between the lower surface of the VAGM and the upper surface of the heat sink. Moreover, due to the limited direct contact area between VAGM and the heat sink (10–30 psi), the upper surface of the heat sink exhibits a significantly non-uniform temperature distribution (Fig. 4g).

For the case of LM-VAGM, when it was in direct contact with the heat sink, the surface-modified liquid metal acts as a buffer layer to connect vertically aligned graphene edges with the paired heat sink. Based on excellent fluidity, liquid metal can fully fill multi-scale voids at the rough heat sink surface under a low packaging pressure (Fig. 4h). As a result, the actual contact area of the “liquid–solid” mating interface formed between the LM-VAGM and the heat sink is much larger than that of the “solid–solid” VAGM/heat sink interface, greatly improving the heat transfer efficiency. In Fig. 4i, the calculated temperature difference between the LM-VAGM and the heat sink is as low as 0.13 °C, with the corresponding effective contact thermal conductance up to 0.982 MW m^−2^ K^−1^, more than an order of magnitude higher than that of the VAGM case (0.059 MW m^−2^ K^−1^). Since the liquid metal buffer layer achieves full contact between the LM-VAGM and the heat source, leading to the significant enhancement of the effective heat transfer area, the temperature distribution at LM-VAGM/heat sink mating interface is much more uniform than that of the VAGM case (Fig. 4g, j). Additionally, in contrast to the pressure-dependent heat transfer efficiency for the case of VAGM, the LM/VAGM/heat sink mating interface achieves decoupling between thermal boundary resistance and pressure, exhibiting almost constant effective contact thermal conductance from 10 to 30 psi. This result indicates that LM-VAGM can maintain relatively constant interfacial heat transfer performance under changing packaging pressure, which is of great significance for improving its application range as a TIM. Based on vertically aligned graphene and surface-modified liquid metal endowing the LM-VAGM with high κ_⊥_ and low *R*_contact_, respectively, the *R*_total_ of LM-VAGM is as low as 9–11 K mm^2^ W^−1^ under a low packaging pressure of 10–30 psi, which is far lower than that of VAGM (59–87 K mm^2^ W^−1^) and comparable to conventional thermal grease.

### TIMs Performance of LM-VAGM

**Fig. 5:**
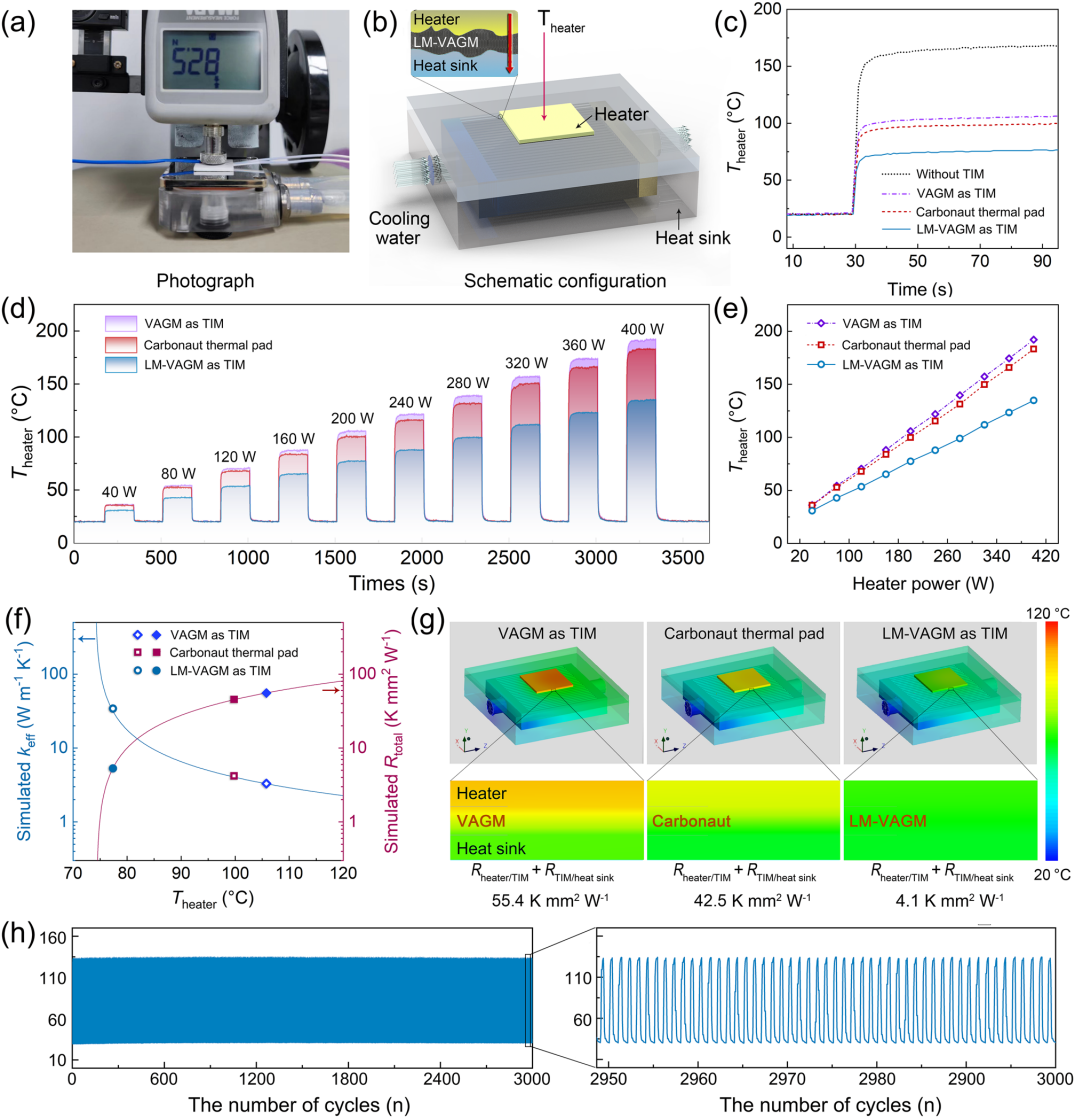
**a** Experimental setup and **b** the schematic configuration of the TIM performance measurement system. **c** *T*_heater_ evolution as a function of heating time at an applied power of 200 W. **d**, **e** The steady-state *T*_heater_ versus the applied power. **f** The simulated κ_eff_ and *R*_total_ of the applied TIM as a function of *T*_heater_. **g** Simulated temperature profiles for cases with VAGM, Carbonaut thermal pad and LM-VAGM as TIMs. **h** Thermal cycling stability of the cooling system with LM-VAGM as TIM in cyclic heating/cooling tests

In order to compare the interface heat transfer efficiency between LM-VAGM and VAGM with TIMs under real packaging conditions, a cooling installation for reducing the operating temperature of high-power electronic devices was set up. As the optical photograph and schematic configuration shown in Fig. 5a, b, respectively, the LM-VAGM and VAGM with the same lateral size of 20 × 20 mm^2^ and a BLT of ≈ 181 μm were mounted between a ceramic heater and microfluidic heat sink under a low package pressure of 30 psi. When the heater was turned on, a microfluidic heat sink was connected to the circulating cooling water to remove the heat generated, and a thermocouple was used to record the time-dependent temperature change of the heater (*T*_heater_). As a comparison, the performance testing under the same conditions for a state-of-the-art commercial TIM based on vertically aligned carbon fiber (Thermal Grizzly Carbonaut thermal pad, 62.5 W m^−1^ K^−1^, Germany) was also performed. In Fig. 5c, when the heater with the applied power of 200 W was directly connected to the microfluidic heat sink without a TIM, the *T*_heater_ raise rapidly as the cooling system started up and reached a steady state of 168 °C at the heating time at 90 s. In contrast, bridging the comparative TIMs between the heater and the heat sink led to a significant decrease in *T*_heater_ (62.2–90.6 °C), suggesting that the TIM plays a key role in the improvement of the cooling efficiency of the system. Noticeably, the best heat dissipation efficiency can be realized by using the LM-VAGM as the TIM, based on the lowest *T*_heater_ of 77.4 °C compared to that of VAGM (105.8 °C) and Carbonaut thermal pad (99.8 °C). Moreover, according to the linear variation of *T*_heater_ with the increase in heating power (Fig. 5d, e), we can calculate the equivalent heat dissipation coefficient (equal to the reciprocal of the slope) of the cooling system for the three cases to be 2.32 (VAGM), 2.46 (Carbonaut thermal pad) and 3.47 W cm^−2^ °C^−1^ (LM-VAGM), respectively. This result indicates that using our LM-VAGM as the TIM achieves 49.8% and 41.1% improvement in heat dissipation efficiency compared to VAGM and Carbonaut thermal pad, respectively.

In order to in-depth study the interfacial heat transfer mechanism of the applied TIMs, we adopted computational fluid dynamics software (Icepak) to calculate the key factors affecting the steady-state temperature distribution of the cooling system (200 W). The detailed model size parameters and material settings were expatiated in Section S5. The simulated evolution curve of the heat transfer resistance from the heater to the heat sink (*R*_toal_) and effective thermal conductivity (κ_eff_) of the applied TIMs versus *T*_heater_ is shown in Fig. 5f. According to the experimental *T*_heater_ in Fig. 5c, we can calculate that the *R*_toal_ and κ_eff_ of LM-VAGM bridged the heater and heat sink is 5.3 K mm^2^ W^−1^ and 34.2 W m^−1^ K^−1^, respectively. This result indicates that the LM-VAGM bridging the heater and heat sink has a significantly higher interface heat transfer efficiency compared to that of VAGM (*R*_toal_ = 55.4 K mm^2^ W^−1^, κ_eff_ = 3.3 W m^−1^ K^−1^) and Carbonaut thermal pad (*R*_toal_ = 45.5 K mm^2^ W^−1^, κ_eff_ = 4.2 W m^−1^ K^−1^). Furthermore, based on the intrinsic κ_⊥_ (bulk thermal conductivity) of the TIM, it can be calculated that the *R*_contact_ (double sides, the sum of *R*_heater/TIM_ and *R*_TIM/heat sink_) of LM-VAGM is as low as 4.2 K mm^2^ W^−1^, which is one order of magnitude lower than that of the other two counterparts (VAGM: 54.3 K mm^2^ W^−1^; Carbonaut thermal pad: 42.5 K mm^2^ W^−1^). This result also indicated that for the TIM with low BLT and high bulk thermal conductivity, the interfacial heat transfer efficiency is almost dominated by the contact thermal resistance, and also demonstrates the critical role of liquid metal surface modification in enhancing the performance of TIMs. By combining high κ_⊥_ and low *R*_total_, the simulated temperature profiles in Fig. 5g intuitively demonstrated the excellent heat dissipation efficiency of the LM-VAGM as TIM. Additionally, toward the practical application, the thermal cycling performance of the LM-VAGM as TIM was investigated by altering the heating power of the heater alternately between 40 and 400 W. The result in Fig. 5h shows that our LM-VAGM can maintain an extremely stable heat dissipation performance during continuous heating/cooling impact for 3000 times.

## Conclusions

In summary, guided by the urgent demands for high-performance TIMs for addressing the thermal management issue of high-power semiconductor devices, we developed a liquid metal-modified vertically aligned graphene monolith (LM-VAGM) by combining mechanical orientation and surface modification. The obtained LM-VAGM has a three-tiered structure composed of mainly vertically aligned graphene in the middle and micrometer-thick liquid metal as a cap layer on the upper and lower surface. Based on the rational structure and surface design, LM-VAGM exhibited an ultra-high through-plane thermal conductivity of 176 W m^−1^ K^−1^ and a low contact thermal resistance of 4–6 K mm^2^ W^−1^. The comparison of actual heat transfer efficiency between the LM-VAGM and state-of-the-art commercial TIMs demonstrated the superior interface heat transfer efficiency of our proposed TIM for cooling electronic devices. This finding not only proposes a design concept for high-performance TIM, but also provides a feasible solution to the common problem of high contact thermal resistance for vertically aligned graphene structures, greatly improving the possibility for the practical application of graphene in electronic thermal management.

### Supplementary Information

Below is the link to the electronic supplementary material.

Supplementary file1 (PDF 1117 kb)
