# Supplementary material for: Ultralow Interfacial Thermal Resistance of Graphene Thermal Interface Materials with Surface Metal Liquefaction
Source: Nanomicro Lett. 2022 Dec 9;15:9. doi: 10.1007/s40820-022-00979-2 (PMC9733747; doi:10.1007/s40820-022-00979-2)
Supplement: Supplementary file 1 — Supplementary file1 (PDF 1117 kb) [file 40820_2022_979_MOESM1_ESM.pdf]

Supporting Information for

# Ultralow Interfacial Thermal Resistance of Graphene Thermal Interface Materials with Surface Metal Liquefaction

Wen Dai[1, 2, #], Xing-Jie Ren[3, #], Qingwei Yan[1, 2, *], Shengding Wang[4], Mingyang Yang[1], Le Lv[1, 2], Junfeng Ying[1, 2], Lu Chen[1, 2], Peidi Tao[1], Liwen Sun[1, 2], Chen Xue[1, 2], Jinhong Yu[1, 2], Chengyi Song[5], Kazuhito Nishimura[6], Nan Jiang[1, 2, *], and Cheng-Te Lin[1, 2, *]

[1] Key Laboratory of Marine Materials and Related Technologies, Zhejiang Key Laboratory of Marine Materials and Protective Technologies, Ningbo Institute of Materials Technology and Engineering (NIMTE), Chinese Academy of Sciences, Ningbo 315201, P. R. China

[2] Center of Materials Science and Optoelectronics Engineering, University of Chinese Academy of Sciences, Beijing 100049, P. R. China

[3] Institute of Advanced Technology, Shandong University, Jinan 250100, P. R. China

[4] Ningbo Institute of Materials Technology and Engineering (NIMTE), Chinese Academy of Sciences, Ningbo 315201, P. R. China

[5] The State Key Laboratory of Metal Matrix Composites, School of Materials Science and Engineering, Shanghai Jiao Tong University, 800 Dong Chuan Road, Shanghai 200240, P. R. China

[6] Advanced Nano-processing Engineering Lab, Mechanical Systems Engineering, Kogakuin University, Tokyo 192-0015, Japan

[#] Wen Dai and Xing-Jie Ren have contributed equally to this work.

*Corresponding authors. E-mail: yanqingwei@nimte.ac.cn (Qingwei Yan); jiangnan@nimte.ac.cn (Nan Jiang); linzhengde@nimte.ac.cn (Cheng-Te Lin)

## Supplementary Table

**Table S1** Comparison of intrinsic through-plane conductivity between LM-VAGM and reported thermally conductive materials based on vertically aligned structure

| Materials | Through-plane *κ* (W $m^{-1}$ $K^{-1}$) | Refs. |
|---|---|---|
| Dense vertically aligned copper nanowire composites | 75 | [S1] |
| Copper–tin nanowire arrays | 98 | [S2] |
| Vertically aligned carbon nano fiber composites | 23.3 | [S3] |
| Vertically aligned carbon nano fiber-based TIM | 24 | HFS-15 |
| Vertically aligned carbon nano fiber | 40 | EX20000 C9 |
| Vertically aligned carbon nano fiber | 62.5 | Carbonaut |
| Vertically aligned multi-walled carbon nanotubes | 27 | [S4] |
| Aligned carbon nanotubes/epoxy composites | 2.7 | [S5] |
| Aligned carbon nanotubes/epoxy composites | 4.87 | [S6] |
| Carbon nanotube arrays/epoxy-DBP film | 17.76 | [S7] |
| Vertical graphene array/PDMS composites | 20.4 | [S8] |
| Vertical graphene array/PDMS composites | 34.2 | [S9] |
| Vertical graphene array/PVDF composites | 12.8 | [S10] |
| Vertically aligned functionalized multilayer graphene | 112.2 | [S11] |
| Vertically aligned high-quality graphene foams/epoxy | 35.5 | [S12] |
| 3D lamellar-structured graphene aerogels/epoxy composite | 20 | [S13] |
| Anisotropic graphene framework/polymer composite | 62.4 | [S14] |
| Vertically oriented graphite/polybutadiene | 64.9 | [S15] |
| Liquid metal surface-modified vertically aligned graphene Monolith | 176 | This work |

## S1 Detailed Characteristic Structure Analysis of the HSGM

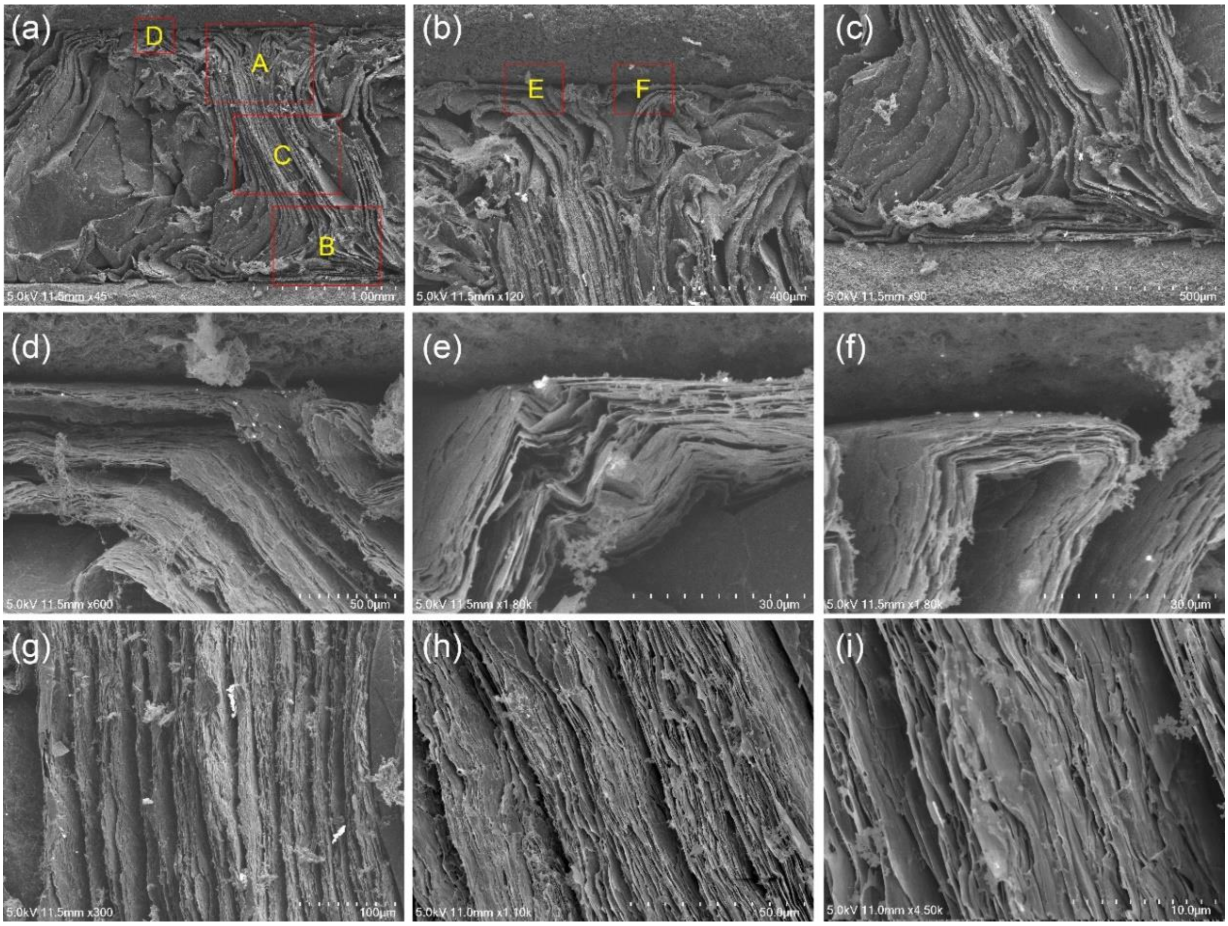


**Fig. S1** A detailed characteristic structure analysis of the HSGM: (**a**) The overall cross-sectional SEM image of HSGM. A partial enlarged view of the (**b**) upper and (**d**) lower surfaces of the HSGM; (**b**) and (**c**) are from positions A and B in **Fig. S1**a, respectively. (**d–f**) Typical cap layer morphology of HSGM; (**d**) and (**e**) are from positions E and F in **Fig. S1**b, respectively, and (**f**) is from position D in **Fig S1**a; (**g–h**) Typical middle layer morphology of HSGM, from position C in **Fig. S1**a

## S2 Detailed Measurement Process of the Thermal Conductivity Based on Laser Flash Method

The thermal conductivity of LM-VAGM was measured by a laser flash method using LFA 467 HyperFlash® system (NETZSCH, Germany). To carry out the measurement, the samples were cut into a square plate with the size of 10 × 10 × 0.8 $mm^3$. All samples are coated by a graphite layer (≈ 5 μm) on both upper and lower sides.

In the actual operation, one side of the sample set in the holder was transiently heated by a xenon lamp pulse with a pulse width of 10 μs, and an infrared radiation detector recorded the rise of surface temperature at another side. The thermal diffusivity was evaluated by analyzing the fitted curve of temperature evolution versus sampling time based on **Eq. S1**:

$$\alpha = 0.1388 \frac{d^2}{t_{50}} \quad \text{(S1)}$$

where $\alpha$, $d$, and $t_{50}$ are the calculated thermal diffusivity, sample thickness, and the half diffusion time, respectively. The $\alpha$ was obtained by measuring three samples separately three times. Accordingly, the thermal conductivity ($\kappa$) could be calculated using **Eq. S2:**

$$\kappa = \alpha \times C_p \times \rho \quad \text{(S2)}$$

where $C_p$ is the specific heat capacity and is measured using LFA 467 with pure graphite as reference (25 °C); $\rho$ is the density of the samples and is calculated from the ratio of mass to volume. The $C_p$ and the $\rho$ were obtained by taking the average value of three separate measurements.

### S2.1 Uncertainty Analysis of LM-VAGM Thermal Conductivity

Based on the measurement principle of the flash method and **Eqs. S1** and **S2**, the uncertainty of the LM-VAGM thermal conductivity mainly comes from the following four factors:

$\mu_1$ — uncertainty calculated from repeated measurements of sample thermal diffusivity ($\alpha_{LM\text{-}VAGM}$) by statistical analysis (A type).

$\mu_2$ — uncertainty introduced by measuring the thickness of a sample (B type).

$\mu_3$ — uncertainty introduced by measuring the specific heat capacity of a sample (B type).

$\mu_4$ — uncertainty introduced by measuring the density of a sample (B type).

### S2.2 Calculation of μ1

**Table S2** The results of ten measurements of thermal diffusivity at 25 °C

| **Number (n)** | 1 | 2 | 3 | 4 | 5 |
|---|---|---|---|---|---|
| **$\alpha_{LM\text{-}VAGM}$ (mm$^2$ s$^{-1}$)** | 369.662 | 369.082 | 368.603 | 368.880 | 365.665 |
| **Number (n)** | 6 | 7 | 8 | 9 | 10 |
| **$\alpha_{LM\text{-}VAGM}$ (mm$^2$ s$^{-1}$)** | 368.527 | 368.591 | 372.676 | 367.682 | 367.632 |

Based on the result in **Table S2**, the standard deviation of $\alpha_{LM\text{-}VAGM}$ can be calculated using **Eq. S3**:

$$s = \sqrt{\frac{\sum_{i=0}^{n}(\kappa_i - \bar{\kappa})^2}{n-1}} = 1.7748\ W\ m^{-1}\ K^{-1} \quad \text{(S3)}$$

Accordingly, the uncertainty calculated from repeated measurements of sample thermal diffusivity can be obtained using **Eq. S4**:

$$\mu_1 = \frac{s}{\bar{\kappa}} = 0.481\% \quad \text{(S4)}$$

### S2.3 Calculation of μ2

The sample thickness was measured by a micrometer and the calibration certificate indicates an uncertainty of 1.3 μm (k = 2). Based on the sample thickness of 800 μm, the relative standard uncertainty ($\mu_{thickness}$) can be obtained:

$$\mu_{\text{thickness}} = \frac{1.3}{2\times800} = 0.081\% \tag{S5}$$

Based on the **Eq. S1**, uncertainty introduced by measuring the thickness of a sample can be obtained by the following:

$$\mu_2 = \sqrt{(\mu_{\text{thickness}})^2 + (\mu_{\text{thickness}})^2} = 0.115\% \tag{S6}$$

### S2.4 Calculation of μ3

Based on the instruction of the instrument, the test accuracy of the specific heat capacity is ±5%. Considering the uncertainty according to the normal distribution, the confidence probability is 99.73%. Therefore, the uncertainty introduced by measuring the specific heat capacity can be obtained:

$$\mu_3 = \frac{5\%}{3} = 1.67\% \tag{S7}$$

### S2.5 Calculation of μ4

The density of the sample is calculated from the ratio of mass (m) to volume (V). The sample mass was measured using analytical balance, with an uncertainty of 0.1 mg (k = 2). Based on the sample mass of 53.6 mg, the relative standard uncertainty ($\mu_{\text{m}}$) can be obtained:

$$\mu_{\text{m}} = \frac{0.1}{2\times53.6} = 0.093\% \tag{S8}$$

The width and length were measured by a micrometer, with an uncertainty of 1.3 μm (k = 2). Based on the sample width/length of 10 mm, the relative standard uncertainty ($\mu_{\text{width/length}}$) can be obtained:

$$\mu_{\text{width/length}} = \frac{1.3}{2\times10000} = 0.0065\% \tag{S9}$$

Accordingly, the uncertainty introduced by measuring the density can be obtained:

$$\mu_4 = \sqrt{(\mu_{\text{m}})^2 + (\mu_{\text{width/length}})^2 + (\mu_{\text{width/length}})^2 + (\mu_{\text{thickness}})^2} = 0.163\% \tag{S10}$$

### S2.6 Calculation of Relative Composite Standard Uncertainty: μe

Based on the above $\mu_1 - \mu_4$, the $\mu_{\text{e}}$ can be calculated:

$$\mu_e = \sqrt{(\mu_1)^2 + +(\mu_2)^2 + (\mu_3)^2 + (\mu_4)^2} = 1.749\% \tag{S11}$$

### S2.7 Calculation of Expanded Uncertainty: Urel

Taking the coverage factor k = 2, $U_{rel}$ can be obtained:

$$U_{rel} = k \times \mu_e = 2 \times 1.749\% = 3.5\% \tag{S12}$$

## S3 Measurement Principle of the TIM Performance Based on ASTM D5470 Method

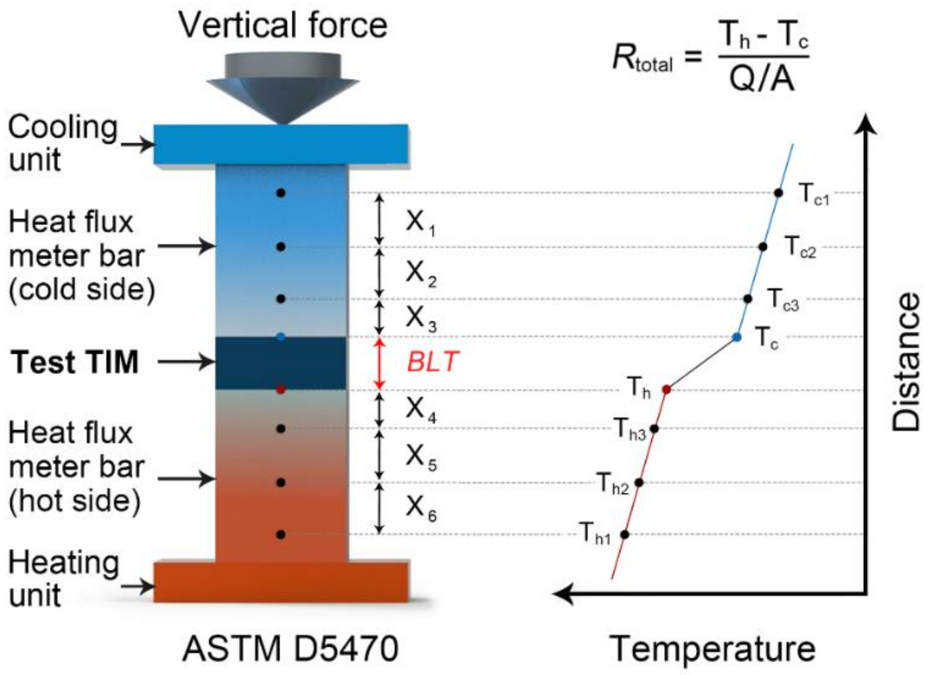


**Fig. S2** Schematic illustrating the measurement principle of the TIM performance based on the modified ASTM D5470 method

The $R_{\text{total}}$ for a TIM was measured using the modified ASTM D5470 method, as schematically illustrated in **Fig. S2**. *BLT* (bond line thickness) is the thickness of test TIM under compression. $X_1$ – $X_6$ is the location of the temperature measurement point on the heat flux meter bar. For the Longwin 9389 equipment, $X_1 = X_2 = X_4 = X_5 = X_6 = 25$ mm; $X_3 = X_4 = 15$ mm. $T_{c1}$ – $T_{c3}$ and $T_{h1}$ – $T_{h3}$ are the measured temperature using six resistance temperature detectors (RIDs). $T_c$ and $T_h$ are the calculated temperature of the upper and lower surfaces of the test TIM, respectively. $Q_c$ and $Q_h$ are the heat flux of the heat meter bar and cold meter bar, respectively. $Q_c$ and $Q_h$ can be extracted from the one-dimensional heat equation, as shown in **Eqs. S13** and **S14:**

$$Q_c = \kappa_{\text{Cu}} \cdot A_{\text{Cu}} \cdot \frac{T_{c1} - T_{c3}}{X_1 + X_2} \tag{S13}$$

$$Q_h = \kappa_{\text{Cu}} \cdot A_{\text{Cu}} \cdot \frac{T_{h1} - T_{h3}}{X_5 + X_6} \tag{S14}$$

where $\kappa_{\text{Cu}}$ and $A_{\text{Cu}}$ are the thermal conductivity and the heat transfer area of the heat flux meter bar, which is made of pure copper, plated with a nanometer-thick layer of nickel. The average heat flux can be calculated using **Eq. S15**:

$$Q = \frac{Q_c + Q_h}{2} \tag{S15}$$

When the test reaches a steady state, **Eqs. S16** and **S17** can be used to describe the relationship between the temperature and the location of the temperature measurement point.

$$\kappa_{Cu} \cdot A_{Cu} \cdot \frac{T_{c3} - T_{c1}}{X_1 + X_2} = \kappa_{Cu} \cdot A_{Cu} \cdot \frac{T_{c3} - T_c}{X_3} \quad (S16)$$

$$\kappa_{Cu} \cdot A_{Cu} \cdot \frac{T_{h1} - T_{h3}}{X_5 + X_6} = \kappa_{Cu} \cdot A_{Cu} \cdot \frac{T_{h1} - T_h}{X_4} \quad (S17)$$

Accordingly, the upper ($T_c$) and lower surfaces ($T_h$) of the test TIM can be obtained based on **Eqs. S18** and **S19**, and the $R_{total}$ can be calculated using **Eq. S20**, where $A_{TIM}$ is the area of the tested TIMs.

$$T_c = T_{c3} - \frac{(T_{c3} - T_{c1}) X_3}{X_1 + X_2} \quad (S18)$$

$$T_h = T_{c1} + \frac{(T_{h1} - T_{h3}) X_4}{X_5 + X_6} \quad (S19)$$

$$R_{total} = A_{TIM} \cdot \frac{T_h - T_c}{Q} \quad (S20)$$

Based on the above principle, we selected three sets of samples with the different original thicknesses (200, 500, and 800 μm) and measured the $R_{total}$ from 10 to 100 psi, as the results shown in **Fig. S3**a, b, respectively. To obtain the $\kappa_{\perp}$ under compression and the corresponding $R_{contact}$, we perform a linear fit to the measured $R_{total}$ versus the *BLT* at each pressure. **Fig. S3**c, d exhibited the typical results for VAGM and LM-VAGM at 30 psi, in which the inverse of the calculated slope is $\kappa_{\perp}$ and the intercept is $R_{contact}$. Based on the same data processing method, we got the $\kappa_{\perp}$ and $R_{contact}$ of VAGM and LM-VAGM from 10 to 100 psi.

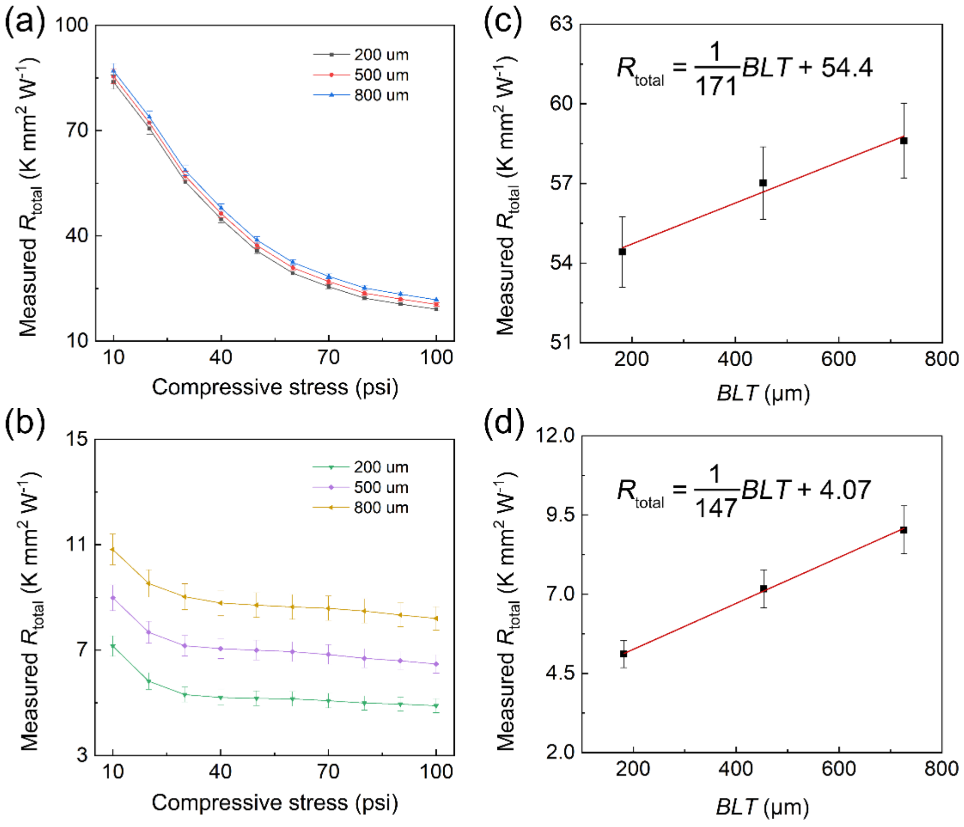


**Fig. S3** The measured $R_{total}$ of (**a**) VAGM and (**b**) LM-VAGM with the original thickness of 200, 500 and 800 μm. The calculation of $\kappa_{\perp}$ of (**c**) VAGM and (**d**) LM-VAGM under the vertical compression of 30 psi based on the measured $R_{total}$

## S3.1 Uncertainty Analysis of the total Resistance of the LM-VAGM

Based on the measurement principle of the ASTM D5470 method using the test equipment of LW 9389 (Long Win), the uncertainty of the total thermal resistance for the LM-VAGM mainly comes from the following three factors:

$\mu_5$ — uncertainty calculated from repeated measurements of sample total thermal resistance ($R_{LM\text{-}VAGM}$) by statistical analysis (A type).

$\mu_6$ — uncertainty introduced by the random error of test equipment (B type).

$\mu_7$ — uncertainty introduced by measuring the lateral size of a sample (B type).

## S3.2 Calculation of μ5

**Table S3** The results of ten measurements of total thermal resistance at 30 psi

| **Number (n)** | 1 | 2 | 3 | 4 | 5 |
|---|---|---|---|---|---|
| **$R_{LM\text{-}VAGM}$ (K mm$^2$ K$^{-1}$)** | 4.012 | 4.141 | 4.066 | 4.021 | 4.081 |
| **Number (n)** | 6 | 7 | 8 | 9 | 10 |
| **$R_{LM\text{-}VAGM}$ (K mm$^2$ K$^{-1}$)** | 4.123 | 4.014 | 4.009 | 4.171 | 4.072 |

Based on the result in **Table S3**, the standard deviation of $R_{\text{LM-VAGM}}$ can be calculated using **Eq. S21**:

$$s = \sqrt{\frac{\sum_{i=0}^{n}(\kappa_i - \bar{\kappa})^2}{n-1}} = 0.059\ K\ mm^2\ W^{-1} \quad \text{(S21)}$$

Accordingly, the uncertainty calculated from repeated measurements of sample thermal conductivity can be obtained:

$$\mu_5 = \frac{s}{\bar{\kappa}} = 1.45\% \quad \text{(S22)}$$

### S3.3 Calculation of μ6

The random error of the test equipment mainly comes from three aspects, including temperature difference measurement error, contact condition error, and instrument accumulated error. According to the device manual (Long Win LW-9389), the values of the three random errors are ±12.0% (temperature difference measurement error), ±3.0% (contact condition error), and ±1.0% (instrument accumulated error), respectively. Treating all these errors as mean distributions, the uncertainty introduced by the random error of test equipment can be obtained:

$$\mu_6 = \sqrt{\left(\frac{12\%}{\sqrt{3}}\right)^2 + \left(\frac{3\%}{\sqrt{3}}\right)^2 + \left(\frac{1\%}{\sqrt{3}}\right)^2} = 7.16\% \quad \text{(S23)}$$

### S3.4 Calculation of μ7

The lateral size (width × length) was measured by a micrometer and the calibration certificate indicates an uncertainty of 1.3 μm (k = 2). Based on the sample width/length of 25.4 mm, the relative standard uncertainty ($\mu_{\text{thickness}}$) can be obtained:

$$\mu_{\text{width/length}} = \frac{1.3}{2\times2540} = 0.026\% \quad \text{(S24)}$$

Accordingly, the uncertainty introduced by measuring the thickness of a sample can be obtained by the following:

$$\mu_7 = \sqrt{(\mu_{\text{width/length}})^2 + (\mu_{\text{width/length}})^2} = 0.037\% \quad \text{(S25)}$$

### S3.5 Calculation of relative composite standard uncertainty: μe

Based on the above $\mu_5 - \mu_7$, the $\mu_e$ can be calculated:

$$\mu_e = \sqrt{(\mu_5)^2 + (\mu_6)^2 + (\mu_7)^2} = 7.305\% \quad \text{(S26)}$$

### S3.6 Calculation of expanded uncertainty: Urel

Taking the coverage factor k = 2, $U_{rel}$ can be obtained:

$$U_{rel} = k \times \mu_e = 2 \times 7.305\% = 14.61\% \quad \text{(S27)}$$

# S4 Thermal Simulation of the TIM/Heat Sink Mating Interface Based on Multi-Block Lattice Boltzmann Method

In order to in-depth elaborate on the interfacial heat transfer process, we used the multi-block lattice Boltzmann method to calculate the temperature distribution of the mating interface formed by a TIM and a heat sink (**Fig. S4**a). The detailed model setup and solution steps are as follows.

## S4.1 Rough Surface Generation

The numerical model is based on the practical topography of the specimens. Firstly, the high-resolution microscope was used to measure the surface topography of the TIM and the heat sink, as the results shown in **Fig. S4**b, c, respectively. The measured area is 5 mm × 5 mm and the unit of height is μm. Then, the measured surface topography data was used to generate the lower surface of the TIM and the upper surface of the heat sink.

It should be noted that the measured data is quite massive that could not be inputted into the software directly, so 1/64 data volume was used to reconstruct rough surfaces. Here, 1/64 means that the used data volume accounts for 1/64 of the original measured data volume. Accordingly, the numerical model of the TIM/heat sink mating interface could be constructed by importing the surface topography data into a pre-processing software named ANSA via the “points→coons surfaces→volume→mesh” flow path. As a result, the coons surface generation was realized through the self-developed Python code read in the ANSA script interface.

**Figure S4**d, e exhibits the imported data points and generated coons surfaces. The distance of every point in both $x$ and $y$ directions is 0.031807 mm. Here, we used 24964 points to generate a rough surface, and the area of every generated rough surface was 4.998 × 4.998 mm$^2$. It can be seen that every small coons surface was generated by four adjacent points, and all small coons surfaces formed an entire continuous rough surface through the “Topo function” of the ANSA software.

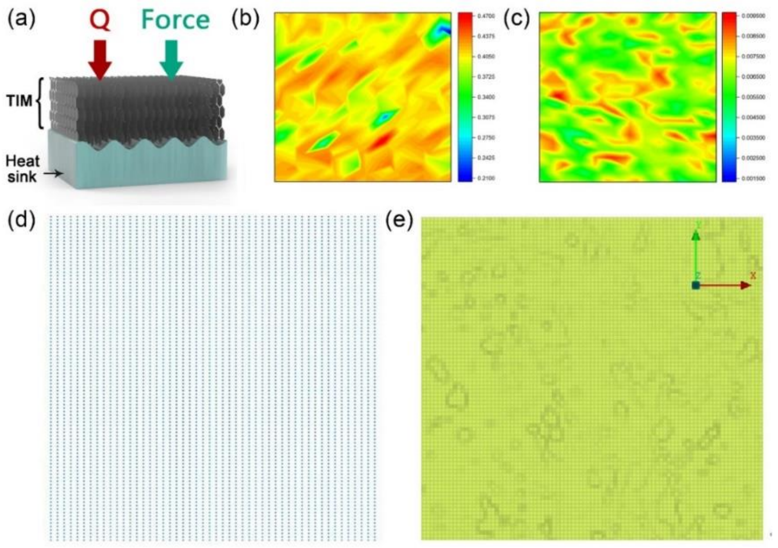


**Fig. S4** (**a**) Schematic illustrating the VAGM as a TIM in contact with the rough surface of the heat

sink. The measured surface roughness of (**c**) VAGM and (**d**) the heat sink. (**d**) The imported data points of the VAGM based on the measured surface roughness, with the reconstructed surface of the TIM shown in (**e**).

### S4.2 Mesh generation

Based on the reconstructed rough surfaces of the TIM and the heat sink, the numerical model of the TIM/heat sink mating interface could be built, as shown in **Fig. S5**a. The height of the TIM and the heat sink are 0.8 mm and 2 mm, and the lateral size of them is 4.998 × 4.998 $mm^2$. When generating meshes, the mating interfaces were meshed firstly, and the imported points were treated as nodes of a single quadrilateral mesh. Then, the quadrilateral meshes were mapped into structured hexahedral meshes. Finally, the whole model has 1469520 structured elements, as the corresponding mating interface shown in **Fig. S5**b.

### S4.3 Materials property settings

The simulation is finished by finite element software ABAQUS. According to the experimental data, the material of the heat sink is set to copper (Cu) with the thermal conductivity of 398 W $m^{-1}$ $K^{-1}$ and the compression modulus of 110 GPa. For the case without liquid metal between TIM and heat sink, we set TIM material properties based on experimental data from VAGM (thermal conductivity: 210 W $m^{-1}$ $K^{-1}$; compression modulus: 2.25 MPa). For the case with liquid metal as a buffer layer, we did not directly use the experimental data of LM-VAGM, but reduced the compressive modulus by 10% based on the material properties of VAGM.

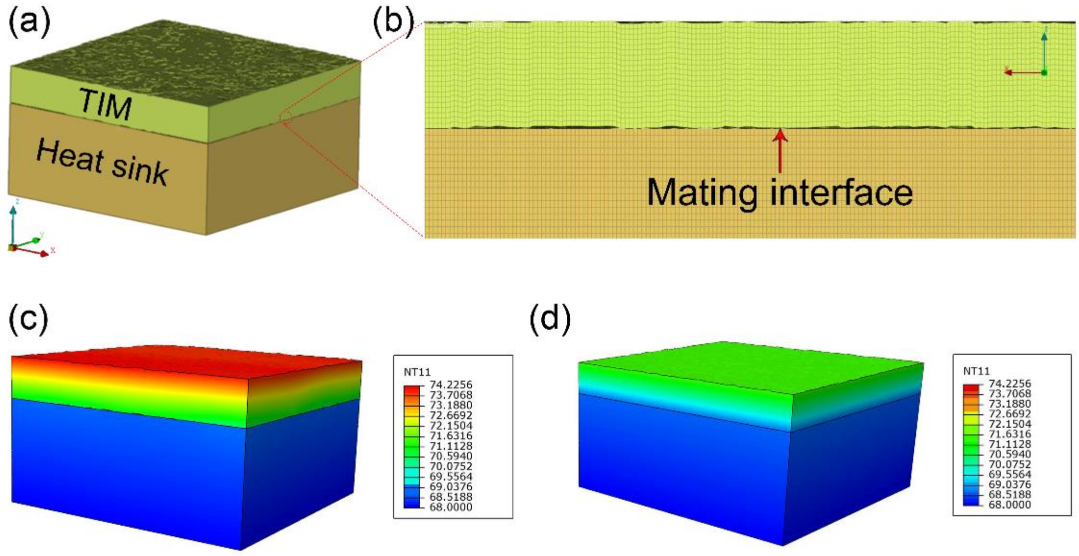


**Fig. S5** (**a**) Schematic illustrating the computational domain of TIM/heat sink model, with the meshes of the corresponding mating interface shown in (**b**). Simulated temperature profiles for (**c**) VAGM and (**d**) LM-VAGM in contact with the heat sink

### S4.4 Boundary conditions and solution

After setting the material properties, the contact computation between the lower surface of the TIM and the upper surface of the heat sink was conducted. In this stage, the thermal expansion between

them was neglected, and different pressures (10, 20, and 30 psi) were loaded on the top surface of TIM. When the contact computation was finished, the actual contact area of the mating interface under different pressure can be obtained. Then, a steady-state heat analysis was conducted on the deformed model. In this stage, a heat flux of 12.4 W $cm^{-2}$ was loaded at the top surface of TIM, and the bottom surface was kept at 68 °C. When the computation converges, we can obtain the temperature distribution of the mating interface, as the results shown in **Fig. S5**c, d, respectively.

## S5 Thermal Simulation of the Thermal Management System Based on CFD Software

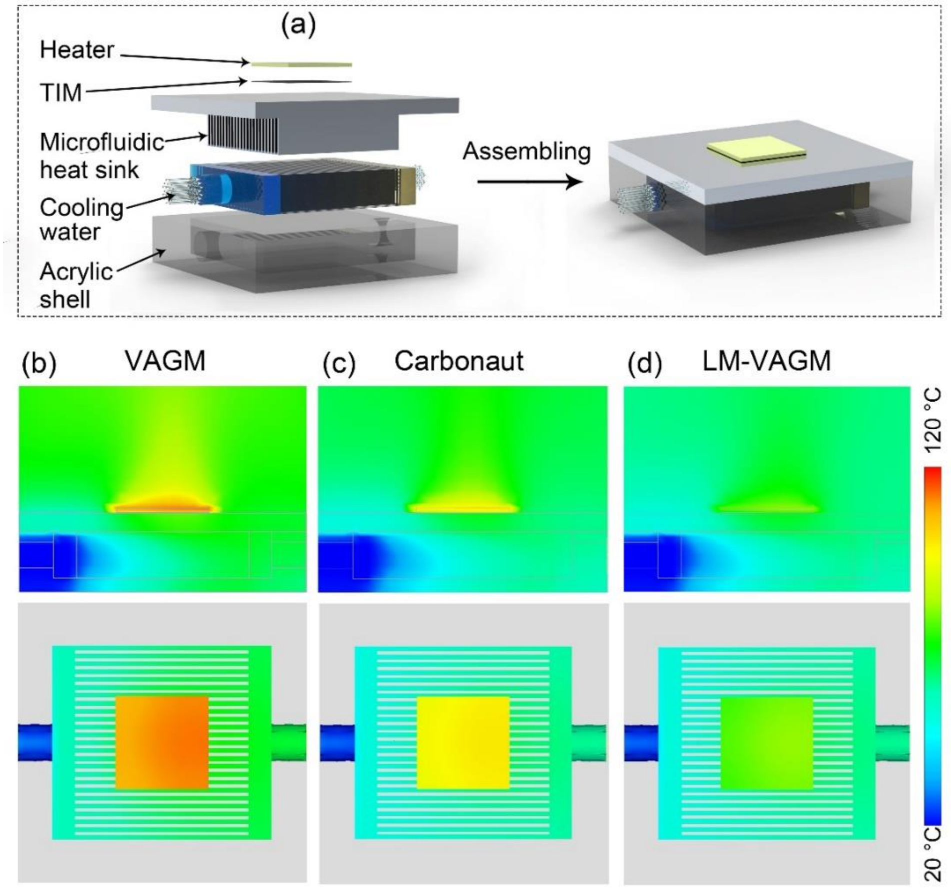


**Fig. S6** (**a**) Icepak system model for TIM performance evaluation. Simulated temperature profiles for cases with (**b**) VAGM, (**c**) Carbonaut thermal pad and (**d**) LM-VAGM as TIMs

In order to in-depth study the TIM performance in an integrated system using LM-VAGM, a commercial computational fluid dynamics software (Icepak) was employed to simulate the heat transfer process in an electronic system with the power of the device (heater) of 200 W. The model implementation is presented in **Fig. S6**a, and the detailed settings of the heater and the heat sink can be found in **Table S4**. When the simulation started, the background temperature was set to 20 °C at 1 atm in the atmosphere, and the entrance temperature of cooling water was 20 °C with a volume flow of 1.26 $cm^3$ $s^{-1}$. **Fig. S6**b-d shows the steady-state temperature profiles of the simulated system with

TIMs, demonstrating a better heat dissipation capability of our LM-VAGM compared to that with VAGM or Carbonaut thermal pad as the TIM. In addition, based on the equation: $R_{contact} = R_{total} - R_{bulk} = BLT/\kappa_{eff} - BLT/\kappa_{bulk}$, the total ($R_{total}$) and contact thermal resistances ($R_{contact}$) of the three TIMs were calculated, with the detailed parameters and results shown in **Table S5**.

**Table S4** The detailed parameters of the components in the simulated system

| | **Size (cm$^3$)** | **Materials** | **$\kappa$ (W m$^{-1}$ K$^{-1}$)** | **$C_p$ (J g$^{-1}$ K$^{-1}$)** |
|---|---|---|---|---|
| Heater | 20 × 20 × 0.1 | Alumina | 27 | 0.91 |
| Heat sink | 62 × 60 × 4.0 | Copper | 398 | 0.39 |
| Fins | 37.2 × 10 × 0.65 | Copper | 398 | 0.39 |
| Shell | 62 × 60 × 13 | Arcylic | 0.2 | 1.17 |

**Table S5** The calculated total thermal resistance ($R_{total}$) and contact thermal resistance ($R_c$) of the three applied TIMs. The *BLT* and the $\kappa_{bulk}$ are the thickness and the thermal conductivity of the samples under packaging conditions

| **Applied TIMs** | ***BLT* (μm)** | **$\kappa_{bulk}$ (W m$^{-1}$ K$^{-1}$)** | **$\kappa_{eff}$ (W m$^{-1}$ K$^{-1}$)** | **$R_{total}$ (K mm$^2$ W$^{-1}$)** | **$R_{contact}$ (K mm$^2$ W$^{-1}$)** |
|---|---|---|---|---|---|
| Without TIM | / | / | / | 229.8 | 229.8 |
| Carbonaut | ≈ 190 | 62.5 | 4.2 | 45.5 | 42.5 |
| VAGM | ≈ 181 | 171.1 | 3.3 | 55.4 | 54.3 |
| LM-VAGM | ≈ 181 | 146.5 | 34.2 | 5.3 | 4.1 |

## Supplementary References


[S1] M.T. Barako, S.G. Isaacson, F. Lian, E. Pop, R.H. Dauskardt et al., Dense vertically aligned copper nanowire composites as high performance thermal interface materials. ACS Appl. Mater. Interfaces **9**(48), 42067-42074 (2017). https://doi.org/10.1021/acsami.7b12313

[S2] W. Gong, P. Li, Y. Zhang, X. Feng, J. Major et al., Ultracompliant heterogeneous copper–tin nanowire arrays making a supersolder. Nano Lett. **18**(6), 3586-3592 (2018). https://doi.org/10.1021/acs.nanolett.8b00692

[S3] K. Uetani, S. Ata, S. Tomonoh, T. Yamada, M. Yumura et al.,. Elastomeric thermal interface materials with high through-plane thermal conducivity from carbon fiber fillers vertically aligned by electrostatic flocking. Adv. Mater. **26**(33), 5857-5862 (2014). https://doi.org/10.1002/adma.201401736

[S4] W. Lin, J. Shang, W. Gu, C.P. Wong, Parametric study of intrinsic thermal transport in vertically aligned multi-walled carbon nanotubes using a laser flash technique. Carbon **50**(4), 1591-1603 (2012). https://doi.org/10.1016/j.carbon.2011.11.038

[S5] W. Lin, K.S. Moon, C.P. Wong, A combined process of in situ functionalization and microwave treatment to achieve ultrasmall thermal expansion of aligned carbon nanotube–polymer nanocomposites: toward applications as thermal interface materials. Adv. Mater. **21**(23),

2421-2424 (2009). https://doi.org/10.1002/adma.200803548

[S6] A.M. Marconnet, N. Yamamoto, M.A. Panzer, B.L. Wardle, K.E. Goodson, Thermal conduction in aligned carbon nanotube–polymer nanocomposites with high packing density. ACS Nano **5**(6), 4818-4825 (2011). https://doi.org/10.1021/nn200847u

[S7] M. Wang, H. Chen, W. Lin, Z. Li, Q. Li et al., Crack-free and scalable transfer of carbon nanotube arrays into flexible and highly thermal conductive composite film. ACS Appl. Mater. Interfaces **6**(1), 539-544 (2014). https://doi.org/10.1021/am404594m

[S8] Q. Yan, F.E. Alam, J. Gao, W. Dai, X. Tan et al., Soft and self-adhesive thermal interface materials based on vertically aligned, covalently bonded graphene nanowalls for efficient microelectronic cooling. Adv. Funct. Mater. **31**(36), 2104062 (2021). https://doi.org/10.1002/adfm.202104062

[S9] S. Xu, T. Cheng, Q. Yan, C. Shen, Y. Yu et al., Chloroform-assisted rapid growth of vertical graphene array and its application in thermal interface materials. Adv. Sci. **9**(15), 2200737 (2022). https://doi.org/10.1002/advs.202200737

[S10] Q. Yan, J. Gao, D. Chen, P. Tao, L. Chen et al., A highly orientational architecture formed by covalently bonded graphene to achieve high through-plane thermal conductivity of polymer composites. Nanoscale **14**(31), 11171-11178 (2022). https://doi.org/10.1039/D2NR02265F

[S11] Q. Liang, X. Yao, W. Wang, Y. Liu, C.P. Wong, A three-dimensional vertically aligned functionalized multilayer graphene architecture: an approach for graphene-based thermal interfacial materials. ACS Nano **5**(3), 2392-2401 (2011). https://doi.org/10.1021/nn200181e

[S12] F. An, X. Li, P. Min, P. Liu, Z.G. Jiang et al., Vertically aligned high-quality graphene foams for anisotropically conductive polymer composites with ultrahigh through-plane thermal conductivities. ACS Appl. Interfaces **10**(20), 17383-17392 (2018). https://doi.org/10.1021/acsami.8b04230

[S13] P. Liu, X. Li, P. Min, X. Chang, C. Shu et al., 3D lamellar-structured graphene aerogels for thermal interface composites with high through-plane thermal conductivity and fracture toughness. Nano-Micro Lett. **13**, 22 (2020). https://doi.org/10.1007/s40820-020-00548-5

[S14] W. Dai, L. Lv, T. Ma, X. Wang, J. Ying et al., Multiscale structural modulation of anisotropic graphene framework for polymer composites achieving highly efficient thermal energy management. Adv. Sci. **8**(7), 2003734 (2021). https://doi.org/10.1002/advs.202003734

[S15] J. Li, Y. Zhang, T. Liang, X. Bai, Y. Pang et al., Thermal interface materials with both high through-plane thermal conductivity and excellent elastic compliance. Chem. Mater. **33**(22), 8926-8937 (2021). https://doi.org/10.1021/acs.chemmater.1c03275
